# Factors Associated with Severe Human Rift Valley Fever in Sangailu, Garissa County, Kenya

**DOI:** 10.1371/journal.pntd.0003548

**Published:** 2015-03-12

**Authors:** A. Desirée LaBeaud, Sarah Pfeil, Samuel Muiruri, Saidi Dahir, Laura J. Sutherland, Zachary Traylor, Ginny Gildengorin, Eric M. Muchiri, John Morrill, C. J. Peters, Amy G. Hise, James W Kazura, Charles H. King

**Affiliations:** 1 Stanford University, Department of Pediatrics, Palo Alto, California, United States of America; 2 UCSF Benioff Children’s Hospital Oakland, Oakland, California, United States of America; 3 Division of Vector Borne and Neglected Tropical Diseases, Ministry of Health, Nairobi, Kenya; 4 Center For Global Health and Diseases, Case Western Reserve University, Cleveland, Ohio, United States of America; 5 Department of Microbiology and Immunology, The University of Texas Medical Branch at Galveston, Galveston, Texas, United States of America; 6 Department of Pathology, Case Western Reserve University, Cleveland, Ohio, United States of America; 7 Research Service, Louis Stokes Cleveland Department of Veterans Affairs, Cleveland, Ohio, United States of America; U.S. Naval Medical Research Unit No. 2, INDONESIA

## Abstract

**Background:**

Mosquito-borne Rift Valley fever virus (RVFV) causes acute, often severe, disease in livestock and humans. To determine the exposure factors and range of symptoms associated with human RVF, we performed a population-based cross-sectional survey in six villages across a 40 km transect in northeastern Kenya.

**Methodology/Principal Findings:**

A systematic survey of the total populations of six Northeastern Kenyan villages was performed. Among 1082 residents tested via anti-RVFV IgG ELISA, seroprevalence was 15% (CI95%, 13–17%). Prevalence did not vary significantly among villages. Subject age was a significant factor, with 31% (154/498) of adults seropositive vs. only 2% of children ≤15 years (12/583). Seroprevalence was higher among men (18%) than women (13%). Factors associated with seropositivity included a history of animal exposure, non-focal fever symptoms, symptoms related to meningoencephalitis, and eye symptoms. Using cluster analysis in RVFV positive participants, a more severe symptom phenotype was empirically defined as having somatic symptoms of acute fever plus eye symptoms, and possibly one or more meningoencephalitic or hemorrhagic symptoms. Associated with this more severe disease phenotype were older age, village, recent illness, and loss of a family member during the last outbreak. In multivariate analysis, sheltering livestock (aOR = 3.5 CI95% 0.93–13.61, P = 0.065), disposing of livestock abortus (aOR = 4.11, CI95% 0.63–26.79, P = 0.14), and village location (P = 0.009) were independently associated with the severe disease phenotype.

**Conclusions/Significance:**

Our results demonstrate that a significant proportion of the population in northeastern Kenya has been infected with RVFV. Village and certain animal husbandry activities were associated with more severe disease. Older age, male gender, herder occupation, killing and butchering livestock, and poor visual acuity were useful markers for increased RVFV infection. Formal vision testing may therefore prove to be a helpful, low-technology tool for RVF screening during epidemics in high-risk rural settings.

## Author Summary

Rift Valley fever virus (RVFV) causes serious disease in both animals and humans. Large-scale outbreaks result in devastating economic losses and create many urgent public health concerns. Among humans, the symptoms of RVF are variable, having a broad spectrum of disease that ranges from mild to severe fever symptoms, and can include ocular complications, encephalitis, and sometimes hemorrhagic disease. In this study, 1082 at-risk Kenyan subjects were serum antibody-tested for evidence of prior RVFV infection and their demographic, health, and exposure data were collated. Seroprevalence was moderately high across the study area (15%) but did not differ significantly among villages across the study region. Age, gender, and herding occupation were all significantly associated with being RVFV seropositive. Older age, village and certain animal husbandry activities were associated with more severe disease. Poor visual acuity was more likely in the seropositive group. This better definition of risk factors and associated symptom complexes should prove helpful for RVF screening during future outbreaks in high-risk rural settings.

## Introduction

Rift Valley fever virus (RVFV) is a mosquito-borne zoonotic disease that poses a significant risk to human health in endemic regions of Africa and the Middle East [1]. Epizootics usually precede epidemics and can result in large-scale abortion storms in local livestock populations [2]. These RVFV outbreaks in human and animal populations result in significant economic damage from trade embargos and significant livestock losses in affected areas [3]. Recent data also demonstrate that RVFV can be transmitted to humans during interepidemic periods [4–6]. RVFV infection is categorized as a neglected tropical disease due to the fact that RVFV disproportionately affects resource-limited semi-nomadic herding communities, is poverty promoting, and has long-lasting sequelae [5]. Additionally, RVF is expanding its range, threatening other areas of the world as an emerging infectious disease; notably, both Europe and the United States have the necessary vectors and livestock reservoirs to sustain autochthonous RVFV transmission [7,8]. The severity of RVFV manifestation, its devastating economic and public health effects, and its potential to be sustained in new regions make the study of RVFV transmission and disease a high priority.

Clinically, most often RVFV causes no symptoms or a mild illness manifesting with fever and liver abnormalities [4]. More rarely, RVFV is known to cause cases of retinitis, encephalitis, or hemorrhagic diathesis with hepatitis during epidemics [9], but these manifestations are variable and currently unpredictable. Most primary infections are thought to cause only self-limited febrile illness followed by complete recovery. It is not yet clear why severe cases occur- these consist of patients with neurologic dysfunction (up to 8%), and hemorrhagic cases (up to 1%, which is then associated with mortality of up to 50%) [2,4]. Furthermore, RVFV causes visual disturbances including reversible anterior uveitis (up to 30% of cases), and permanent retinitis (up to 20%) [10]. This broad spectrum of human RVF disease has been most recently confirmed in investigations of the 2006–2007 epidemics in East Africa [9]. Outbreaks in NE Kenya (Garissa County) were reported during the last epidemic [2], but RVFV activity in nearby Ijara constituency (Masalani and Sangailu), was not specifically monitored. Other reports have shown evidence of interepidemic human RVFV transmission in Ijara constituency (Masalani)[5–6]. It has been suggested that clinical phenotype of disease may be in part determined by the route of RVFV transmission, with animal-related transmission likely to be more severe than mosquito borne disease [1]. To expand this knowledge, the goal of the present study was to identify the exposures and other risk factors associated with human RVFV transmission and disease severity in a typical East African endemic setting, the Ijara constituency, Sangailu location, Kenya.

## Methods

### Ethics statement

All participants provided written consent under a protocol approved by the Human Investigations Review Board of University Hospitals Case Medical Center (No. 11–09–01) and the Ethical Review Committee of the Kenya Medical Research Institute, Nairobi, Kenya (Non-SSC Protocol No. 195). Before participation, written informed consent was obtained from adult study subjects, and parents provided written informed consent for their participating children. Children over 7 years of age also provided individual assent.

### Location

**Fig 1:**
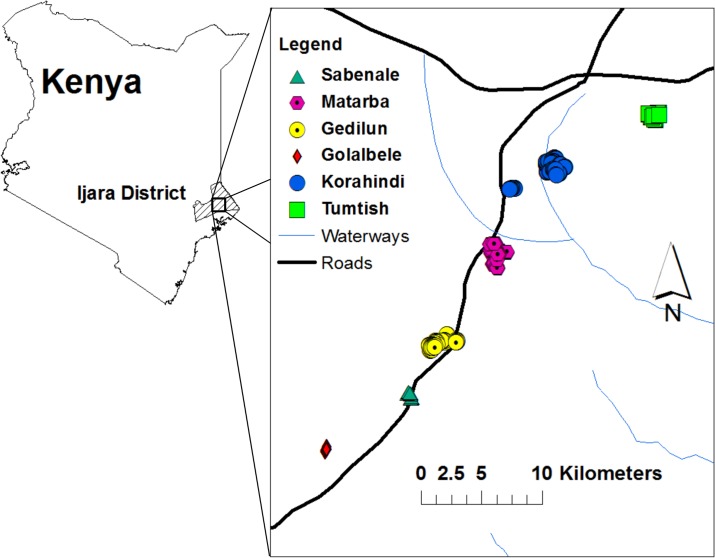
Map showing the location of the Sangailu study area in Northeastern Province in Kenya. The country map, left, indicates the boundaries of Ijara District, the health administration unit at the time of the study (the Sangailu location is now part of Garissa County, Ijara constituency). The inset, right, shows the locations of the six community clusters that were included in the study. These were distributed in a 40 km transect along a main road of the Sangailu area.

**Fig 2:**
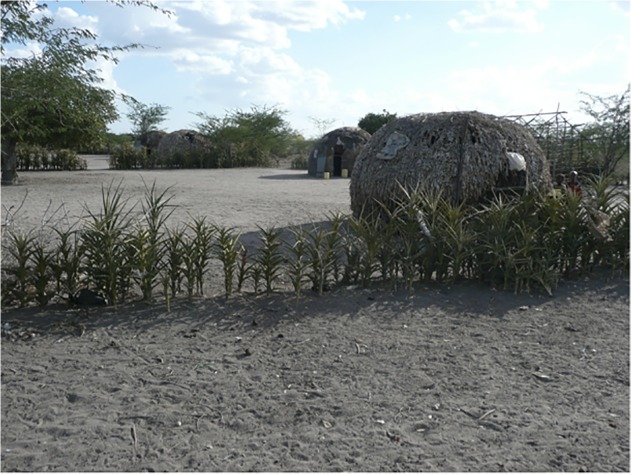
Typical peri-domestic landscape in the Sangailu area of Kenya. Shown are traditional wicker and grass-mat domed houses and outbuildings, surrounded by a planted compound perimeter and local dense brush vegetation.

This study was performed in the semi-arid Sangailu Location of Ijara constituency, Kenya. Six villages (Golabele, Sabenale, Gedilun, Matarba, Korahindi, and Tumtish) were sampled for demographic, epidemiological, and health information during area-wide household surveys performed from August through November of 2011, five years after the last known RVF epidemic in the area (2006–2007). The villages are located off a main road across a span of approximately 40 Km, in a transect running southwest to northeast (Fig. 1) centered around coordinates 1 deg. 19 min S, 40 deg. 44 min E. The northern-most village, Tumtish, is located 39 Km from the border with Somalia. The participating populations studied were comprised predominantly of herders and semi-nomadic pastoralists of Somali ethnicity. A typical household landscape is shown in Fig. 2. For unique identification and subsequent analysis of the spatial distribution of RVFV serostatus, participating household locations were geo-referenced by Global Positioning System with the use of a Garmin eTrex handheld device (Garmin, Schaffhausen, Switzerland).

### Participants

Study recruitment began after consultation and approval by local leaders and administrators. After an initial demographic census was performed to determine the current local population and its distribution, a systematic survey of the total populations of six Northeastern Kenyan villages was performed. The villages were systematically surveyed in sequence to reach the desired sample size of >1000 enrolled individuals. All residents were eligible for inclusion, except that those residing in the area for <2 years, and children less than 1 year of age were excluded. The study sample was representative of the local ethnic mix of 99% Kenyan Somali and < 1% Bantu, Indian, or other Asian.

### Examination procedures

Participants had formal interviews to detail their demographics, occupation, mosquito exposure, and animal exposure within the last 6 months, and any previous nonspecific symptoms related to RVF in the last 30 days, or severe symptoms at any time (see Supporting S1 Text). Visual acuity testing by use of the Snellen chart and physical exams were performed, with an emphasis on signs of recent or remote RVF. Children under 5 yrs. old (N = 358) were excluded from visual acuity testing. Poor visual acuity was defined as a score less than or equal to 6/9 meters in either eye by standard Snellen eye chart testing. A stratified subset (118) of visually symptomatic and asymptomatic subjects also underwent dilated fundoscopic eye exam and imaging using a retinal camera. Peripheral blood was then collected for serological testing for anti-RVFV IgG.

### Laboratory testing

Sera were tested for IgG against RVFV by standardized ELISA protocol [11,12] and confirmed by plaque reduction neutralization testing (PRNT), as previously described [5,6,12–14]. Specimens having an ELISA OD of >0.25 and a PRNT titer of ≥1:20 were considered positive. The confirmatory plaque reduction neutralization testing (PRNT) was performed at the University of Texas, Medical Branch at Galveston.

### Statistical methods

Statistical analysis examined the association of subject demographic and exposure factors with two primary outcomes: i) odds of seropositivity and ii) odds of having had the more severe symptoms of RVF. Initial chi-square tests were performed to identify the association of categorical factors with RVFV seropositivity and t-tests were used for continuous variables. A series of nested multivariable logistic regression models were next developed that initially included predictors significant in bivariate comparisons, as well as those considered of biological relevance prior to conduct of the study. Bivariate results and stepwise regression models were used to aid in the selection of variables to be included in the final models. Non-significant variables (p > = 0.10) were removed in stepwise fashion to help identify the variables with the greatest multiply adjusted links to RVFV seropositivity or symptom score. For this analysis, statistical significance was set at the 0.05 level. Following analysis of individual symptoms, a relative RVF severity score was developed using the two-step cluster algorithm in SPSS v. 21 (IBM, Armonk NY, USA) to empirically define significant constellations of milder, moderate, and more severe symptom states among RVFV seropositive subjects [15]. The severe disease phenotype was defined as having somatic symptoms of acute fever plus eye symptoms and possibly one or more meningoencephalitic or hemorrhagic symptoms (S1 Fig). Mild disease phenotype was defined as RVFV seropositivity with few to no symptoms. Multivariate logistic regression models were run using severe *vs*. mild disease categories and significant variables from bivariate analysis, excluding those variables used to define disease severity. These statistical models were performed using SAS software (SAS Institute Inc. version 9.3, Cary, NC, USA).

Statistical analysis of spatial patterns of seropositivity among the participating households was performed with the use of Point Pattern Analysis software [16] and Clusterseer 2.0 software (Biomedware, Ann Arbor, MI)

## Results

### Associations with RVFV seropositivity

Of the 1134 participants enrolled in the study, 1082 completed all phases of the examination and were tested for RVFV infection. Of these, 164 were RVFV seropositive (15%; CI_95%_ 13–17%). Males were more likely to be RVFV infected: 18% (79/433) were seropositive compared to 13% (85/646) of females (*P* = 0.023; Table 1). Adults (≥16 years old) were also more likely than children to be RVFV infected: Thirty-one percent (152/487) of adults were seropositive compared to 2% (12/595) of children (*P* < 0.001). The average age of seropositive people was 42 ± 19.5 years (range 6–85 years) *vs*. 17 ± 17 for seronegatives (range 1–84 years). No significant differences in seropositivity were seen among the six villages studied in the Sangailu region: Golabele (17.6%; 15/85), Korahindi (17.0%; 49/288); Sabenale (15.6%; 10/64); Matarba (14.3%; 33/231); Gedilun (13.9%; 32/230), and Tumtish (13.6%; 25/184). This was not surprising given the uniformity of landscape and environmental features, and the socioeconomic homogeneity within pastoralist communities of this region.

**Table 1:** Results of bivariate analysis to determine the significant relative odds of having anti-RVFV seropositivity according to demographic data, clinical signs, symptoms, and exposure factors.

Significant Variables	Number tested (N)**	Variable Present (N)	Variable Absent (N)	Variable Missing	Odds Ratio (95% Confidence Interval)	P- Value†
Age (continuous)	1134	1082	0	0	1.06 (1.05–1.07)	<0.0001
Sex (Male as reference)	1129	431	646	5	1.45 (1.07–2.08)	0.023
Herder Occupation	1134	122	960		4.95 (3.25–7.54)	<0.0001
Poor Visual Acuity*	1134	111	599	372	5.08 (3.30–7.82)	<0.0001
Recent^^^ Sickness	1132	548	533	1	1.95 (1.37–2.77)	0.0001
Recent^^^ Backache	1132	480	601	1	6.76 (4.48–10.20)	<0.0001
Recent^^^ Nausea	1132	290	791	1	2.03 (1.42–2.90)	<0.0001
Recent^^^ Malaise	1132	356	725	1	3.87 (2.69–5.55)	<0.0001
History of^<^ Severe Bruising	1132	5	1076	1	8.99 (1.48–54.51)	0.005
History of^<^ Red Eyes	1132	146	935	1	4.05 (2.70–6.06)	<0.0001
History of^<^ Poor Vision	1132	112	969	1	4.71 (3.01–7.36)	<0.0001
History of^<^ Eyes Sensitive to Light	1132	159	922	1	4.20 (2.71–6.51)	<0.0001
History of^<^ Eye Pain	1132	164	917	1	5.47 (3.66–8.19)	<0.0001
History of^<^ Spinning Feeling	1132	164	917	1	3.13 (2.09–4.69)	<0.0001
History of^<^ Mental confusion	1132	11	1070	1	10.19 (2.90–35.73)	<0.0001
History of^<^ Sleepy Feeling	1134	64	1017	1	1.89 (1.04–3.43)	0.024
History of^<^ Stiff neck	1132	24	1057	1	5.61 (2.39–13.14)	<0.0001
History of^<^ Seizures	1132	6	1075	1	5.91 (1.17–29.80)	0.017
Personal history of RVF	1132	11	1070	1	4.81 (1.45–16.01)	0.005
Sheep contact	1130	959	118	5	2.57 (1.24–5.34)	0.014
Goat contact	1130	959	118	5	2.57 (1.24–5.34)	0.014
Cow contact	1130	855	221	6	3.06 (1.74–5.40)	<0.0001
Sheltered livestock	1130	709	370	3	6.10 (3.59–10.35)	<0.0001
Sheltered sheep	1130	698	381	3	5.95 (3.54–9.99)	<0.0001
Sheltered goat	1130	704	375	3	5.82 (3.46–9.80)	<0.0001
Sheltered cow	1130	620	459	3	4.94 (3.15–7.76)	<0.0001
Killed livestock	1130	352	727	3	5.50 (3.65–8.30)	<0.0001
Killed sheep	1130	328	751	3	4.35 (2.90–6.52)	<0.0001
Killed goat	1130	290	789	3	3.53 (2.31–5.39)	<0.0001
Killed cow	1130	271	859	4	2.87 (1.85–4.45)	<0.0001
Butchered livestock	1130	510	569	3	5.20 (3.44–7.85)	<0.0001
Butchered sheep	1130	476	603	3	3.39 (2.32–4.96)	<0.0001
Butchered goat	1130	387	692	3	2.36 (1.62–3.43)	<0.0001
Butchered cow	1130	338	741	3	1.74 (1.18–2.56)	0.004
Handled raw meat	1130	886	193	3	2.32 (1.33–4.05)	0.004
Consumed sheep meat	1130	882	197	3	2.40 (1.38–4.19)	0.003
Consumed goat meat	1130	880	199	3	2.43 (1.39–4.24)	0.002
Consumed cow meat	1130	805	274	3	1.94 (1.24–3.04)	0.005
Milked livestock	1130	708	371	3	4.92 (2.97–8.14)	<0.0001
Milked sheep	1130	699	380	3	4.61 (2.83–7.51)	<0.0001
Milked goat	1130	737	393	4	4.88 (2.98–8.01)	<0.0001
Milked cow	1130	606	473	3	4.79 (3.08–7.43)	<0.0001
Cared for birthing animal	1130	632	447	3	5.88 (3.66–9.46)	<0.0001
Birthed sheep	1130	627	452	3	6.07 (3.77–9.77)	<0.0001
Birthed goat	1130	630	449	3	6.35 (3.92–10.31)	<0.0001
Birthed cow	1130	499	580	3	6.02 (3.97–9.15)	<0.0001
Disposed of livestock fetus	1130	608	471	3	5.31 (3.39–8.32)	<0.0001
Disposed of sheep fetus	1130	604	475	3	5.16 (3.31–8.04)	<0.0001
Disposed of goat fetus	1130	607	472	3	5.38 (3.43–8.42)	<0.0001
Disposed of cow fetus	1130	479	600	3	5.05 (3.38–7.54)	<0.0001
Recent family death	1132	65	1016	1	3.22 (1.84–5.64)	<0.0001

† As determined by bivariate Χ^2^ testing

*Poor visual acuity defined functionally at >6/9 meters using a Snellen eye chart

^^^ “Recent” defined as symptoms reported in the last 30 days

^<^ “History of” defined as symptom reported at any time

** N = number of participants with data collected in survey

From initial bivariate analysis, RVFV seropositivity was significantly associated with multiple environmental exposures, as well as certain physical signs and reported symptoms (see Table 1). After multivariable adjustment, our most parsimonious logistic model of seropositive status found that older age (4% increase per year CI_95%_ 2–9%, p<0.0001), male gender (adjusted Odds Ratio (aOR) = 1.8, CI_95%_ 1.2–2.7, *P* < 0.01), poor measured visual acuity (aOR = 1.7, CI_95%_ 1.01–3.0, *P* < 0.05), a history of malaise (aOR = 1.6, CI_95%_ 1.06–2.5, *P* < 0.03), and a history of killing livestock (aOR = 2.0, CI_95%_ 1.4–3.3, *P* < 0.001) were each independently associated with seropositivity (Table 2).

**Table 2:** Significant associations for RVFV seropositivity identified by multivariable logistic regression modeling.

Variables	Variable Present (N)	Variable Absent (N)	Variable Missing (N)	Multiply-adjusted Odds Ratio (95% Confidence Interval)	P Value
Age (continuous)	1082	0	0	1.04 (1.02–1.05)	<.0001
Sex (female as reference)	431	646	5	1.71 (1.13–2.60)	0.012
Poor* visual acuity	111	599	372	1.71 (0.99–2.96)	0.054
Killed livestock	352	727	3	1.98 (1.31–3.00)	0.001
Model R^2^ = 0.25, Akaike’s information criterion (AIC) = 634.6; Condition index 10.6					

*Poor visual acuity defined at less than or equal to 6/9

Ten percent (112/1081) of people surveyed self-reported poor vision. Those who were RVFV-infected were more likely to report poor vision: 26% (43/164), as compared to 7.5% (69/917) among uninfected (*P* < 0.0001). Sixteen percent (111/710) of tested subjects had poor measured visual acuity. Those who were anti-RVFV positive were more likely to have poor measured visual acuity, 36% (57/159) compared to 9.8% (54/551) of seronegatives (*P* < 0.0001). Fundoscopic exams were performed on 118 study participants. Here, objective eye disease was defined as having uveitis, retinitis, retinal scar or retinal hemorrhage. Overall, 26% (30/118) were found to have eye disease defined as retinitis or retinal hemorrhage. Of those serologically tested for RVFV seropositivity, 21% (6/28) were RVFV seropositive.

**Fig 3:**
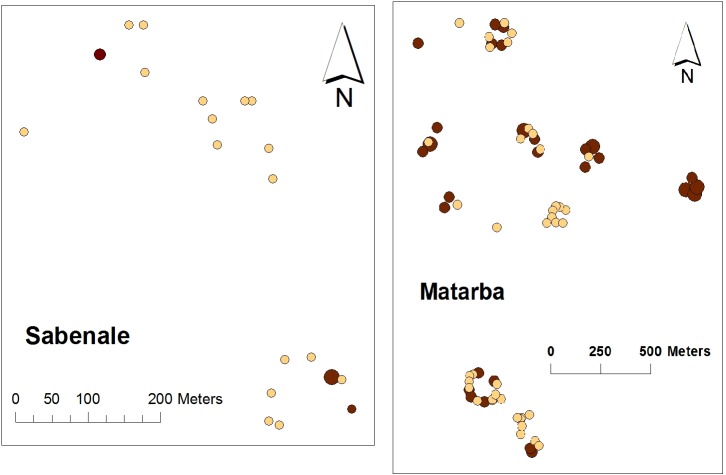
Map of the distribution of RVFV seropositives for study villages Sabenale and Matarba. Within each village, circles represent the location of study subjects’ households. Darker shaded circles indicate houses with RVFV seropositive residents. Larger circles indicate that > 50% of residents were seropositive.

Across the study landscape, we did not find any significant *global* pattern of clustering for anti-RVFV serostatus beyond the underlying distribution of households in each village (using Ripley’s weighted K-function testing [17] over a range from 50 to 850 meters). However, within Korahindi, Matarba, Tumtish, and Sabenale there was evidence (using the Getis G-statistic [18]) of significant *local* clustering, at the 25–100 m scale, of greater per-household density of cases within the certain sections of these communities. Most remarkably, it was noted that all of the confirmed RVFV-positive subjects in Sabenale came from just 3 of 20 houses sampled (Fig. 3, left panel). One of these three seropositive houses in Sabenale had 7 seropositive people out of 11 total household residents. Fig. 3‘s right panel indicates the clustering pattern within the Sangailu area’s central village of Matarba.

### Associations with RVF disease severity

One hundred sixty-four people were RVFV exposed and included in this analysis. Ninety-three percent (152/164) were adults and 52% (85/164) were female. Thirty percent (49/164) were from Korahindi, 20% (33/164) from Matarba, 20% (32/164) from Gedilun, 15% (25/164) from Tumtish, 9% (15/164) from Golalbele, and 6% (10/164) from Sabenale. Those in the moderate/severe group (N = 111) were more likely to be older (p = 0.007; mean age 44.9 years vs. 36.13 years) than those in the mild group (N = 53). In bivariate analysis, the most severe of the RVF symptom-cluster phenotypes was associated with older age, village, recent illness, and death of a family member (Table 3). In a multivariable logistic model controlling for age and village, it was found that those who sheltered livestock or disposed of livestock fetuses were at significantly greater risk for having this more severe illness complex (Table 4). Sheltering livestock put one at three and a half times the risk for more severe disease (aOR = 3.5 CI_95%_ 0.93–13.61, *P* = 0.064). Disposing of livestock abortus put one at four times risk of having a severe disease phenotype (aOR = 4.11, CI_95%_ 0.63–26.79, *P* = 0.140).

**Table 3:** Results of bivariate analysis to determine significant relative odds of high RVF disease severity according to demographic data, clinical signs, symptoms, and exposure factors.

Variable	Variable Present (N)	Variable Absent (N)	Variable Missing (N)	Odds Ratio (95% Confidence Interval) for more severe disease	P Value†
Age (continuous)	164	0	0	1.03 (1.01–1.04)	0.007
Adults (≥16 years old) vs. Children	152 (adults)	12 (kids)	0	2.23 (0.69–7.29)	0.205
Gender (male as reference)	79 (male)	85 (female)	0	0.85 (0.44–1.63)	0.738
Village	164	0	0		0.010
Gedilun				2.10 (0.72–6.12)	
Golalbele				1.94 (0.48–7.86)	
Matarba				0.85 (0.34–2.14)	
Sabenale				4.36 (0.51–37.48)	
Tumtish				0.32 (0.12–0.88)	
Korahindi (ref)					
Herder occupation	48	116	0	0.72 (0.36–1.46)	0.366
Father deceased	94	70	0	1.84 (0.95–3.57)	0.091
Mother deceased	68	96	0	1.41 (0.72–2.78)	0.397
Recent illness	106	58	0	3.87 (1.94–7.72)	.0001
Personal history of RVF	5	159	0	0.71 (0.12–4.37)	0.659
Sheep contact	154	8	2	0.59 (0.12–2.97)	0.280
Goat contact	154	8	2	0.59 (0.12–2.97)	0.280
Cow contact	148	14	2	0.76 (0.23–2.50)	0.108
Camel contact	34	122	8	1.13 (0.49–2.58)	0.956
Sheltered livestock	143	20	1	1.90 (0.73–4.92)	0.204
Sheltered sheep	142	21	1	1.73 (0.68–4.40)	0.316
Sheltered goat	142	21	1	1.73 (0.68–4.40)	0.316
Sheltered cow	133	30	1	1.08 (0.47–2.52)	0.832
Killed livestock	97	66	1	0.88 (0.45–1.73)	0.736
Killed sheep	87	76	1	0.97 (0.50–1.88)	1.00
Killed goat	73	90	1	0.82 (0.43–1.59)	0.614
Killed cow	62	101	1	0.87 (0.44–1.70)	0.730
Butchered livestock	124	39	1	1.09 (0.51–2.34)	0.845
Butchered sheep	109	54	1	1.10 (0.55–2.21)	0.859
Butchered goat	84	79	1	1.09 (0.57–2.11)	0.867
Butchered cow	67	96	1	1.04 (0.53–2.04)	1.00
Handled meat	147	16	1	0.46 (0.13–1.70)	0.275
Consumed livestock	34	129	1	0.51 (0.23–1.11)	0.100
Consumed sheep	109	54	1	0.69 (0.21–2.24)	0.859
Consumed goat	147	16	1	0.69 (0.21–2.24)	0.778
Consumed cow	136	27	1	0.70 (0.28–1.80)	0.508
Milked an animal	143	20	1	0.90 (0.33–2.50)	1.000
Milked sheep	141	22	1	0.77 (0.28–2.11)	.0806
Milked goat	142	21	1	0.84 (0.30–2.29)	0.807
Milked cow	133	30	1	0.74 (0.30–1.78)	0.665
Drank animal milk	159	4	1	2.18 (0.30–15.92)	0.593
Drank sheep milk	160	3	1	1.07 (0.09–12.06)	1.000
Drank goat milk	161	2	1	2.16 (0.13–35.17)	0.538
Drank cow milk	158	5	1	1.44 (0.23–8.89)	0.654
Birthed an animal	140	23	1	0.72 (0.27–1.95)	0.633
Birthed sheep	140	23	1	0.55 (0.19–1.57)	0.337
Birthed goat	141	22	1	0.59 (0.21–1.69)	0.461
Birthed cow	125	38	1	0.84 (0.38–1.85)	0.696
Disposed of fetus	136	27	1	0.88 (0.36–2.17)	1.000
Disposed of sheep fetus	135	28	1	1.01 (0.42–2.42)	1.000
Disposed of goat fetus	136	27	1	0.88 (0.36–2.17)	1.000
Disposed of cow fetus	118	45	1	1.09 (0.53–2.27)	0.852
Mosquito net use	33	131	0	0.68 (0.31–1.49)	0.405
Recent mosquito bites	13	151	0	1.08 (0.32–3.68)	1.000
Mosquito control	4	160	0	1.44 (0.15–14.22)	1.000
Lost Family member	22	142	0	5.60 (1.26–24.95)	0.013

† As determined by bivariate Χ^2^ testing

**Table 4:** Significant associations for greater RVF disease severity determined by multivariable logistic regression modeling.

Variable	Variable Present (N)	Variable Absent (N)	Variable Missing (N)	Multiply-adjusted Odds Ratio (95% Confidence Interval)	P Value
Age (continuous)	163	0	1	2.72 (0.74–9.95)	0.133
Village (Korahindi as reference village)	163	0	1	Gedilun 1.98 (0.64–6.16)	0.009
				Golalbele 1.84 (0.41–8.24)	
				Matarba 1.25 (0.41–3.86)	
				Sabenale 7.34 (0.74–72.73)	
				Tumtish 0.25 (0.09–0.72)	
Disposed of fetus	136	27	1	4.11 (0.63–26.79)	0.140
Birthed livestock	140	23	1	0.17 (0.02–1.43)	0.104
Sheltered livestock	143	20	1	3.55 (0.93–13.61)	0.065
Model R^2^ = 0.22, Akaike’s information criterion (AIC) = 196.22; Condition index 12.0; Hosmer and Lemeshow p = 0.50					

## Discussion

A significant proportion of the population in the semi-arid areas of northeastern Kenya have been infected with RVFV. Other than older age, most of the factors significantly associated with anti-RVFV seropositivity and RVF disease severity were related to pastoralist lifestyles and animal exposures. These included the common practices of livestock shelter at home and livestock fetus disposal, as typically observed in this region. During epizootics, RVFV-infected herds will experience abortion storms and affected virus-contaminated abortus is often handled by herders, significantly increasing their risk for RVFV infection by aerosol and direct contact, and possibly, their risk for more severe RVF disease [1]. Similarly, infected animals brought to slaughter provide potential avenues for transmission via direct blood contact or aerosolization. Animal husbandry exposures were similar in each of the studied villages, which may explain the lack of difference in seropositivity among villages. No empiric global clustering effect was observed for household anti-RVFV seroprevalence across the study landscape, but within some villages, significant local clustering of seropositive households was documented and severe disease manifestations were more common in certain villages than others. It seems that, within certain communities, a few high-risk households carry the burden of RVFV infection, perhaps defined by combined eco-social landscape factors. Between the identified high-risk households, differences in animal husbandry practices could not be determined, but their (unmeasured) animal herd seropositivity could have differed, leading to greater individual household exposures. Other factors such as socioeconomic status and local landscape (vegetation and soil) may have also played a role in exposure risk variation as seen in the last RVFV Kenyan outbreak [19], but these were not measured in our study.

As noted in other studies, RVFV seropositivity rates were much lower among children [5,6,12]. Although there is a built-in age/time bias, in that older people have had a longer time to be exposed to RVFV infected mosquitoes and livestock, the present study and past studies suggest a significant step in RVFV infection risk over the age of 15 years [5,6]. Cultural practices may have limited children’s exposure, as they may be less likely to directly handle infectious materials before the age of 16 years.

Of the subject symptoms we elicited, backache was the most strongly associated with RVFV seropositivity. Among confirmed, hospitalized RVF patients, an initial syndrome consisting of severe headache, fever, arthralgias, and general malaise has been described that occurs prior to the onset of delirium and mental confusion and/or hemorrhagic manifestations [9]. Among encephalitis-related symptoms, photophobia, mental confusion, and meningismus were all associated with evidence of past RVFV infection (Table 1). This is significant because longitudinal case-series in West Africa have noted that RVF may result in long-term schizophrenic or dementia-like manifestations [20]. Our observed association between mental confusion symptoms and RVFV seropositivity is consistent with this previously documented RVFV-related finding.

Self-reported visual impairment and reduced measured visual acuity were both correlated with RVFV seropositivity. Uveitis and/or retinitis are two of the most common sequelae of human RVF [6]. In our study, a history of eye pain, red eyes, or photophobia (eyes sensitive to light) were significantly associated with RVFV seropositivity, which may have been due to RVF uveitis at the time of acute infection. Of the subset of subjects who had retinitis on fundoscopic examination, only one quarter were seropositive, demonstrating a larger burden of unrelated retinal disease in this community.

The analysis of RVF disease severity (Tables 3 and 4) suggests that exposure factors have only a minimal impact on the risk for disease severity, even though they increase risk for infection (as seen in Table 1). This agrees with a recent paper from Anyangu et al [1], in which four exposure factors were associated with severe disease versus nonsevere RVF disease during bivariate analyses (animal contact/herding animals, caring for animals during birthing, touching an aborted animal fetus, and being a herdsperson); however, only one factor (touching an aborted animal fetus) was associated with disease severity in the multivariable model. Our study found that sheltering livestock and disposal of a livestock fetus were associated with severity of disease, but these were not statistically significant in our model. The lack of statistical significance may be due to the small study number (164) in this analysis and could be affected by the remote timing of our study in relation to the last outbreak. It is likely that individual level factors (genes, co-morbidities) also determine risk of disease severity, and only not mode of infection.

This study was limited by the self-reported nature of the exposures and symptom data, which are subject to recall bias. Other prevalent infections, such as malaria, may have accounted for the reported fever-related symptoms. In particular, other arbovirus infections, such as West Nile virus, chikungunya virus, and dengue, might have accounted for reported ocular symptoms, as they, too, are known to cause uveitis and retinitis [21–23]. Because those local residents who had experienced severe RVFV disease in 2006–2007 could have had up to a 50% chance of dying, a survival bias is inherent in our study, and the factors associated with the most severe RVFV disease phenotype (hemorrhagic fever and death) may not have been identified. Also we performed our analysis of severe disease with a small sample size of only 164 participants. This limited sample size may have prevented the elucidation of some factors associated with severe RVF disease.

In conclusion, RVFV infection in northeastern Kenya is significantly associated with older age, male gender, livestock harvesting, and poor vision. Spatial analysis suggests that very high-risk households exist within at-risk communities, which appear to harbor most of the RVFV infection burden. Animal exposure factors were linked to severity of human RVF disease symptoms, as suggested in previous studies [1]. Finally, the prominence of vision-related symptoms and ocular findings suggests that these may prove to be useful indicators of active or recent RVF disease in at-risk settings where serological or PCR RVFV testing is not available.

## Supporting Information

S1 Checklist STROBE Checklist. (DOC)

S1 Text Survey questionnaire used for recording exposures and past symptoms related to RVF. (PDF)

S1 Fig Seropositive subjects’ mild, moderate, and severe symptom clusters, as empirically defined by SPSS two-step cluster algorithm. The relative weights for presence or absence of a given symptom used in the classification are indicated in the left-hand panel. As indicated the right side columns, subjects classified as *severe* RVF had multiple eye-related symptoms plus systemic symptoms of acute febrile illness. Those with *moderate* RVF reported systemic symptoms, but not eye complaints. Subjects with mild disease had seropositivity but reported no symptoms. Ten additional symptoms were scored and entered into the analysis, but did not differ among the three groups.
(TIFF)

S1 Dataset De-identified dataset used for all analysis presented in this manuscript. (XLS)
