# Supplementary material for: Factors Associated with Severe Human Rift Valley Fever in Sangailu, Garissa County, Kenya
Source: PLoS Negl Trop Dis. 2015 Mar 12;9(3):e0003548. doi: 10.1371/journal.pntd.0003548 (PMC4357470; doi:10.1371/journal.pntd.0003548)
Supplement: S1 Text — (PDF) [file pntd.0003548.s002.pdf]

MRCE-CWRU-DVBND RVF surveys: Part 1, PERSONAL DETAILS

ID [ ] Date of Registration (dd/mm/yyyy) [ ]

Subject's First Name [ ]
Second Name [ ]
Third Name [ ]
Other name [ ]

House Number [ ]
Village [ ]

Head of Household's Name [ ]
Relationship to HH [ ]
Usual Occupation [ ]
Did you SLEEP in this house LAST NIGHT? ○ Yes ○ No
Year of Birth [ ]
Sex (F=Female, M=Male) [ ]
How many YEARS living here?: [ ] Stay how many MONTHS next year? [ ]

Father's name: [ ]
Is father living? ○ Yes ○ No ○ Don't Know
Where does he live? [ ]
F's father: [ ]
F's mother: [ ]

Mother's name: [ ]
Is mother living? ○ Yes ○ No ○ Don't Know
Where does she live? [ ]
M's father: [ ]
M's mother: [ ]

Demography team to check village records and complete boxes below:

F study number: [ ]
F house number: [ ]
M study number: [ ]
M house number: [ ]

Name of Data Collector [ ]
Name of Data Enterer [ ]

Principal Investigators: LaBeaud, Kazura, King, Muchiri

MRCE-CWRU-DVBND RVF surveys: Part 2, NON-ANIMAL EXPOSURES

ID [ ] IF NOT HOUSEHOLD INFORMANT, skip this page, go to Part 3

Has your home been flooded in past 4 yrs? ○ Yes ○ No

If yes, when was it flooded? (year) [ ]

Have you ever been displaced by floods? ○ Yes ○ No

If yes, when were you displaced? (year) [ ]

Do you get mosquito bites frequently? ○ Yes ○ No

Do you avoid mosquito bites? ○ Yes ○ No

IF NO, skip to Part B, below

Do you sleep under a mosquito net? ○ Yes ○ No

Do you use mosquito coil? ○ Yes ○ No

If yes, how often do you use coils? [ ]

Indicate: Never, Other, Monthly, Weekly, or Daily

Do you use other mosquito control? ○ Yes ○ No

If yes, what kind? [ ]

Do you have screens on your home windows? ○ Yes ○ No

Part B:

Do you get mosquito bites during the daytime? ○ Yes ○ No

Do you get mosquito bites during the nighttime? ○ Yes ○ No

When was the last time you felt unwell? [ ]

Indicate: < 1month, 1-3 mo, 4-6mo, 7-12mo, 1-2 yr, or never

Have you had a death in the family in last 4 yrs? ○ Yes ○ No

If yes, how many?: [ ]

If yes, when?: [ ]

Notes: [ ]

What is your source of drinking water? Mark all that apply.

River	○ Yes ○ No	Inside water container	○ Yes ○ No
Pond or Pool	○ Yes ○ No	Kiosk or Tanker	○ Yes ○ No
Rain or cistern	○ Yes ○ No	Water tap/piped	○ Yes ○ No
Public well/borehole	○ Yes ○ No	Other	○ Yes ○ No

Interviewer: note type of roofing.

Indicate: natural material, corrugated metal or plastic, or other

Interviewer: note type of latrine.

Indicate: bush, pit, VIP latrine, toilet, other

Interviewer: note type of flooring.

Indicate: dirt, wood, cement, tile, or other

List objects around home that collect water [tires, pots, plants,etc.]

ID

How often do you have sheep contact?:

Indicate: Never, Other, Monthly, Weekly, or Daily

How often do you have goat contact?:

Indicate: Never, Other, Monthly, Weekly, or Daily

How often do you have cow contact?:

Indicate: Never, Other, Monthly, Weekly, or Daily

How often do you have camel contact?:

Indicate: Never, Other, Monthly, Weekly, or Daily

Have you SHELTERED livestock in your home? ○ Yes ○ No

camel ☐ sheep ☐ goat ☐ cow ☐ other ☐

Do you SLAUGHTER livestock? ○ Yes ○ No

If yes, when was the last time?

camel ☐ sheep ☐ goat ☐ cow ☐ other ☐

Do you SKIN or BUTCHER livestock? ○ Yes ○ No

If yes, when was the last time?

camel ☐ sheep ☐ goat ☐ cow ☐ other ☐

Do you eat RAW MEAT? ○ Yes ○ No

Do you HANDLE RAW MEAT in cooking? ○ Yes ○ No

camel ☐ sheep ☐ goat ☐ cow ☐ other ☐

Have you ever MILKED an animal? ○ Yes ○ No

camel ☐ sheep ☐ goat ☐ cow ☐ other ☐

Have you ever DRUNK RAW MILK? ○ Yes ○ No

camel ☐ sheep ☐ goat ☐ cow ☐ other ☐

Do you ASSIST DURING the birth of livestock? ○ Yes ○ No

camel ☐ sheep ☐ goat ☐ cow ☐ other ☐

Have you DISPOSED OF AN ABORTED ANIMAL FETUS? ○ Yes ○ No

camel ☐ sheep ☐ goat ☐ cow ☐ other ☐

MRCE-CWRU-DVBND RVF surveys: Part 4, PATIENT SYMPTOMS

ID ______

Are you sick now? ○ Yes ○ No

How long have you been sick?: ______

Have you had any of these symptoms in the LAST 30 DAYS?

Fever	○ Yes ○ No	No appetite	○ Yes ○ No
Backache	○ Yes ○ No	Vomiting	○ Yes ○ No
Nausea	○ Yes ○ No	Malaise	○ Yes ○ No

Have you EVER had any of these symptoms?

Vomiting Blood	○ Yes ○ No	Severe bruising for no reason	○ Yes ○ No
Red eyes	○ Yes ○ No	Gum bleeding	○ Yes ○ No
Poor vision	○ Yes ○ No	Painful eyes to light	○ Yes ○ No
Eye pain	○ Yes ○ No		
Spinning feeling because of an illness	○ Yes ○ No	Mental Confusion because of an illness	○ Yes ○ No
Much too sleepy because of an illness	○ Yes ○ No	Severe neck stiffness	○ Yes ○ No
Coma	○ Yes ○ No	Seizures or Fits	○ Yes ○ No

Principal Investigators: LaBeaud, Kazura, King, Muchiri

MRCE-CWRU-DVBND RVF surveys: Part 5, PHYSICAL EXAM

ID ____ Weight in kg ____ Height in cm ____

Wasted? ○ Yes ○ No

Scleral Hemmorhages? ○ Yes ○ No

Scleral Icterus? ○ Yes ○ No

Jaundice? ○ Yes ○ No

Petechiae? ○ Yes ○ No

Liver Enlarged? ○ Yes ○ No

Spleen Enlarged? ○ Yes ○ No

Lymphadenopathy ____

Comments ____

Name of Medical Doctor ____

Name of Data Enterer ____

Principal Investigators: LaBeaud, Kazura, King, Muchiri

MRCE-CWRU-DVBND RVF surveys: Part 6, OPHTHALMOLOGIC EXAM

ID

Visual Acuity-L

Visual Acuity-R

Anterior Chamber-L

Anterior Chamber-R

Anterior Uveitis-L? Yes No

Anterior Uveitis-R? Yes No

Posterior Chamber-L

Posterior Chamber-R

Vitreous reaction-L? Yes No

Vitreous reaction-R? Yes No

Retina-L

Retina-R

Retinitis-L? Yes No

Macular-L? Yes No

Paramacular-L? Yes No

Retinitis-R? Yes No

Macular-R? Yes No

Paramacular-R? Yes No

Retinal Hemorrhage-L? Yes No

Zone-L

Area-L

Retinal Hemorrhage-R? Yes No

Zone-R

Area-R

Optic disc edema-L? Yes No

Retinal vasculitis-L? Yes No

Optic disc edema-R? Yes No

Retinal vasculitis-R? Yes No

Comments: (Is this RVF-related or Other?)

Ophthalmologist Name

Data Enterer Name

Principal Investigators: LaBeaud, Kazura, King, Muchiri
