# Supplementary figures and images for: Factors Associated with Severe Human Rift Valley Fever in Sangailu, Garissa County, Kenya
Source: PLoS Negl Trop Dis. 2015 Mar 12;9(3):e0003548. doi: 10.1371/journal.pntd.0003548 (PMC4357470; doi:10.1371/journal.pntd.0003548)

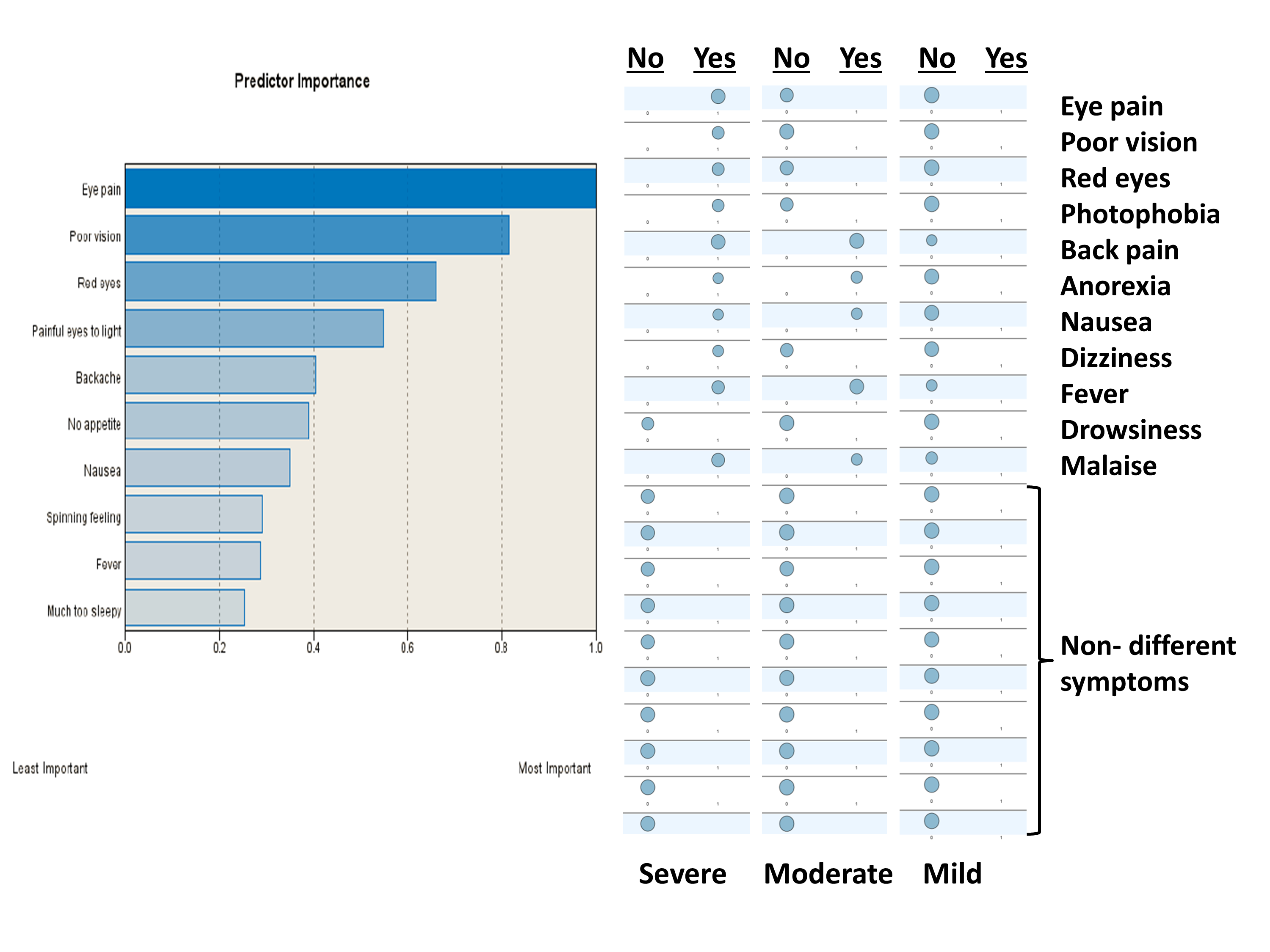

Supplement: S1 Fig — The relative weights for presence or absence of a given symptom used in the classification are indicated in the left-hand panel. As indicated the right side columns, subjects classified as severe RVF had multiple eye-related symptoms plus systemic symptoms of acute febrile illness. Those with moderate RVF reported systemic symptoms, but not eye complaints. Subjects with mild disease had seropositivity but reported no symptoms. Ten additional symptoms were scored and entered into the analysis, but did not differ among the three groups. (TIFF) [file pntd.0003548.s003.tiff]
